# Neutrophils exhibit an individual response to different oral bacterial biofilms

**DOI:** 10.1080/20002297.2020.1856565

**Published:** 2020-12-09

**Authors:** Carina Mikolai, Katja Branitzki-Heinemann, Alexandra Ingendoh-Tsakmakidis, Meike Stiesch, Maren von Köckritz-Blickwede, Andreas Winkel

**Affiliations:** a Department of Prosthetic Dentistry and Biomedical Materials Science, Hannover Medical School, Hanover, Germany; b Department of Physiological Chemistry, and Research Center for Emerging Infections and Zoonoses (RIZ, University of Veterinary Medicine Hannover, Hanover, Germany

**Keywords:** NETs, neutrophils, ROS, periodontitis, oral innate immunity, *Streptococcus oralis*, *Aggregatibacter actinomycetemcomitans*, *Porphyromonas gingivalis*

## Abstract

Oral innate immunity is led by neutrophils. It is still unclear how their main antimicrobial mechanisms against different biofilms may contribute to balance or dysregulation in the oral cavity. We investigated the capacity of commensal (*Streptococcus oralis*) and pathogenic (*Porphyromonas gingivalis* or *Aggregatibacter actinomycetemcomitans*) monospecies biofilms to induce or to inhibit selected antimicrobial mechanisms of neutrophils. *S. oralis* induced neutrophil extracellular traps (NETs) formation, reactive oxygen species (ROS) production, and matrix metalloproteinases (MMPs) 8 and 9 secretion. However, these responses were partially reduced in PMA-activated neutrophils indicating a balance-like neutrophil response, which might be important for the maintenance of oral health. *P. gingivalis* generally induced ROS. Reduced NET formation and significantly decreased MMP secretion were detectable in activated neutrophils highlighting *P. gingivalis’* nucleolytic and proteolytic activity, which might support bacterial colonization and pathogenesis of periodontitis. In contrast, *A. actinomycetemcomitans* did not affect the levels of antimicrobial factors in activated neutrophils and induced NET formation, ROS production, and secretion of MMP-8 and -9 in neutrophils alone, which might contribute to tissue destruction and disease progression. In summary, neutrophil responses to biofilms were species-specific and might support either maintenance of oral health or pathogenesis of periodontitis depending on the species.

## Introduction

The oral microbiome consists of several hundred different bacterial species [1–4]. These are able to form three-dimensional structures with an extracellular polymeric matrix, known as ‘biofilms’ [3–6]. Bacteria within biofilms change their gene expression compared to the planktonic state and are more resistant to antimicrobial agents as well as to the host’s protective immune response [5,7–9]. The oral health-associated multispecies biofilm consists mostly of *Streptococcus* spp. and *Actinomyces* spp., where *Streptococcus oralis* dominate the early formation stage [10–12]. A shift in the composition of the biofilm with higher proportion of pathogenic bacteria (i.e. *Porphyromonas ginigivalis, Aggregatibacter actinomycetemcomitans*) can lead to periodontitis, which has a high prevalence and is a chronic multifactorial inflammatory disease [10,13,14]. Until 2018, periodontitis was divided into an aggressive and a chronic form, among others. Now, both forms are combined under a single category. Periodontitis is defined on the basis of a stage and grade system, while ‘grade’ describes the risk or evidence of the progression rate [14,15]. *P. gingivalis* and *A. actinomycetemcomitans* are convincingly implicated in this progression [16] and *A. actinomycetemcomitans* is often associated with an aggressive and rapid progression rate [17,18].

The main innate immune cell types in the oral cavity are polymorphonuclear neutrophils, which constantly migrate towards a chemotactic gradient of host-derived and microbial chemoattractants through the junctional epithelium – in particular in response to an inflammatory signal. They form a robust antimicrobial barrier, act as the first line of host defense against the oral microbiome, and play an important role in the maintenance of oral health as well as in the pathogenesis of periodontitis [17,19–22].

Neutrophils are the predominant effector cells that respond to periodontal bacteria through a variety of antimicrobial mechanisms, such as phagocytosis, degranulation of antimicrobial proteins, or neutrophil extracellular traps (NETs) formation (NETosis), which are closely associated with the production of reactive oxygen species (ROS) [23–25]. NETs have been found in the gingival crevicular fluid and in purulent exudates from periodontal pockets [26,27]. They are released into the extracellular environment to entrap and immobilize bacteria and thus prevent bacterial spreading and colonization [23,28]. NETs consist of DNA as the core element decorated with a huge array of antibacterial compounds, such as histones, various enzymes – including neutrophil elastase and myeloperoxidase (MPO), as well as antimicrobial peptides [24,28,29]. It has been suggested that exaggerated NETosis and the subsequent release of destructive proteins participates in the local breakdown of connective tissue leading to detrimental effects – including periodontal tissue destruction [28,30]. NETs are more detectable in periodontitis than in oral health [31].

Generation of ROS is a distinct strategy to kill bacteria intracellularly in phagolysosomes or to attack, e.g. biofilms extracellularly [23]. ROS are key components needed to efficiently fight microbes; however, ROS can directly cause tissue damage by lipid peroxidation, DNA and protein damage, and oxidation of enzymes. Although ROS can be neutralized by antioxidants, an imbalance between ROS and antioxidants generated through an excessive oxidative burst leads to ROS-mediated periodontal tissue damage [32].

In addition, neutrophils have different types of granules, which release antibacterial proteins, either into phagolysosomes or into the extracellular space [24]. Matrix metalloproteinase 8 and 9 (MMP-8, MMP-9) are included in the specific or gelatinase granules of neutrophils, respectively [33,34]. They degrade structural components of the extracellular matrix and thus accelerate neutrophil availability at inflammatory sites and also influence several other physiological processes (e.g. antibacterial defense, tissue homeostasis, and remodeling, cell migration and immune cell activation) [35]. On the other hand, their elevated expression leads to tissue destruction [36]. It has been shown that MMP-8 and -9 are the predominant MMPs and the most important enzymes that control the pathogenesis of periodontitis [36,37]. However, it is known that the total absence of MMP-8 results in extensive progression of periodontitis [38] so that it appears that a physiological MMP-level contributes to the resolution of inflammation [35].

Neutrophils play an essential role in the host response to oral biofilm [22,23]. The balance of neutrophil reaction and the constitutive neutrophil apoptosis with removal of these cells are important for maintaining oral health; correspondingly, the imbalance can lead to the development of periodontitis [21,39]. On the one hand, hyperactive neutrophils and the local accumulation of destructive enzymes released by neutrophils, such as MMPs or ROS, can lead to tissue destruction, which contributes to disease progression. On the other hand, neutrophil deficiency and their reduced antimicrobial responses may also contribute to the progression rate of periodontitis [23,40,41]. Neutrophils are able to react specifically to various bacterial species [42]. However, little is known about how the different neutrophil responses to the various bacterial species in the multispecies biofilm might contribute to periodontitis or to the maintenance of oral health. Most of the previous studies used planktonic bacteria or just their lipopolysaccharides (LPSs) as well as other pathogen-associated molecular patterns (PAMPs), which do not represent the common situation in the oral cavity [30,42–48]. Hence, the knowledge about how the main antimicrobial mechanisms, which could be implicated in tissue destruction, are affected in neutrophils by commensal and pathogenic periodontal bacteria that grow as biofilms is important to further understand the role of neutrophils in maintaining oral health or in the pathogenesis of oral diseases [44]. Therefore, on the one hand, the bacterial species-specific NETs formation of neutrophils, their ROS production as well as MMP-8 and -9 secretion in responses to commensal (*S. oralis*) and pathogenic (*P. gingivalis* or *A. actinomycetemcomitans*) monospecies biofilms were investigated. On the other hand, it was determined if the monospecies biofilms were able to inhibit selected antimicrobial mechanism of PMA-stimulated neutrophils.

## Materials and methods

### Biofilm formation

*S. oralis* (ATCC®9811, American Type Culture Collection) was cultivated in tryptone soya broth medium supplemented with 10% yeast extract (TSBY) under aerobic conditions at 37°C. For biofilm formation, *S. oralis* was diluted in TSBY supplemented with 50 mM glucose (TSBYG) to an optical density (OD_600 nm_) of 0.05, approximately corresponding to 2 × 10^10^ CFU/mL. *P. gingivalis* (DSM 20709, German Collection of Microorganism and Cell Cultures) was cultivated in brain heart infusion (BHI) medium supplemented with 10 µg/mL vitamin K under anaerobic conditions (80% N_2_, 10% H_2_, 10% CO_2_) at 37°C. For biofilm formation, *P. gingivalis* was diluted in fresh BHI supplemented with 10 µg/mL vitamin K to OD_600 nm_ = 0.01, approximately corresponding to 7.38 × 10^4^ CFU/mL. *A. actinomycetemcomitans* JP2 strain (HK1651, CCUG56173, Culture Collection University of Gothenburg) isolated from aggressive juvenile periodontitis was cultivated in Todd-Hewitt broth supplemented with 10% yeast extract (THBY) at 37°C in a humidified atmosphere with 5% CO_2_. For biofilm formation, the smooth morphotype of *A. actinomycetemcomitans* was diluted in fresh THBY to OD_600 nm_ = 0.3, approximately corresponding to 1.73 × 10^7^ CFU/mL. All biofilms were cultivated either on glass coverslips (12 mm in diameter) in a 24-well plate, or in a 96-well plate for 24 h under appropriate conditions. Samples of each biofilm were exemplarily stained with LIVE/DEAD® BacLight^TM^ Bacterial Viability Kit (Life Technologies) and analyzed by confocal laser scanning microscope (CLSM, TCS SP8, Leica) as previously described [49].

### Neutrophil isolation and cultivation with biofilm

Human peripheral blood was drawn from four healthy donors with the approval of Hannover Medical School Ethics Committee (Ethics Statement No. 3295–2016). Polymorphnuclear leucocytes which mainly consisted of neutrophils (approx. 95%) [50] were isolated by density gradient centrifugation using PolymorphPrep (Axis-Shield, Oslo, Norway), as previously described [51]. Neutrophils were then resuspended in RPMI 1640 without supplements (PAA, Freiburg, Germany) to a concentration of 1 × 10^6^ cells/mL.

Neutrophils were

- cultivated with each single biofilm to analyze selected antimicrobial responses specifically induced by the individual biofilms: biofilms were washed with phosphate-buffered saline solution (PBS) before neutrophils were added on the biofilms (24-well plate: 5 × 10^5^ cells in 500 µL, 96-well plate: 1 × 10^5^ cells in 100 µL) and cultivated for 3.5 h at 37°C in a humidified atmosphere with 5% CO_2_.
- stimulated with 25 nM phorbol 12-myristate 13-acetate (PMA) [52], which was added immediately before co-incubation with individual biofilms, in order to investigate the potential of the bacteria to inhibit antimicrobial mechanisms of neutrophils.
- cultivated without biofilm (with or without PMA) in the same experimental setup in order to serve as controls.

### NET visualization

After neutrophil incubation with the respective biofilm, NET formation regarding their structure associated with the biofilm was visualized by immunocytochemistry (ICC) or scanning electron microscope (SEM). For ICC, the cells were fixed with 4% paraformaldehyde (PFA). NET visualization was performed as previously described [53]. In short, after permeabilization and blocking, NETs were primary stained with a rabbit polyclonal anti-human MPO antibody (1:300 in blocking buffer, Dako A0398). The secondary staining was performed using a goat anti-rabbit Alexa Fluor 568-conjugated antibody (1:500 in blocking buffer, Thermo Scientific A11031). The samples were embedded in ProlongGold® antifade with DAPI (P36931, Molecular Probes) and analyzed by CLSM (TCS SP5, Leica). For SEM, the cells were fixed with 0.1% glutaraldehyde and 4% PFA diluted in 200 mM HEPES (4-(2-hydroxyethyl)-1-piperazineethanesulfonic acid). The samples were dried and sputtered as previously described [54] and analyzed by SEM (Crossbeam 540, Zeiss). Both techniques allowed the qualitative observation of NETs.

### Nuclease activity assay

The supernatants were collected after neutrophil incubation with the respective biofilm. The nuclease activity of the supernatants was measured using a DNA molecular beacon as previously described, but with slight modifications [55]. In short, the palindrome DNA molecular beacon (5′-FAM-CGA ATT CCT TTT TGG AAT TCG-Quencher-3′, Eurofins genomics) was used at a concentration of 0.1 µmol/L in the reaction buffer (50 mM TRIS-HCl, 5 mM CaCl_2_, 100 µg/mL BSA, pH 7.9). The supernatants were added and the fluorescence (excitation: 485/20 nm, emission: 528/20 nm) was determined every 2 min for 90 min at 37°C.

### ROS assay

ROS production of neutrophils in response to the different biofilms was determined in 96-well plates. After 30 min of neutrophil activation, 2′,7′-dichlorofluorescein diacetate (DCF-DA) was added to a final concentration of 10 µM. The amount of ROS was measured every 30 min over a time period of 3 h (excitation: 485 nm, emission 520 nm).

### Quantification of matrix metalloproteinase

Matrix metalloproteinase 8 and 9 (MMP-8, MMP-9) were measured in the collected supernatants using enzyme-linked immunosorbent assays (ELISAs, R&D systems). ELISAs were performed according to the manufacturer’s protocol. The concentrations of MMP-8 and -9 were calculated using four-parameter logistic (4-PL) equation resulting from the standard curve.

### Statistical analyses

All presented data were derived from four donors cultivated with four independent biofilms of each species. The statistical analysis was performed using GraphPad Prism 8.0. Statistical differences of nuclease activity compared to *Staphylococcus aureus* nuclease knock-out strain (Sa^nuc) were analyzed using one-way analysis of variance (ANOVA) with Bonferroni correction. Differences between the groups regarding ROS production or MMP secretion were analyzed using two-way ANOVA with Bonferroni correction. Statistical differences were considered significant at p < 0.05.

## Results

The isolated human neutrophils were incubated with the different monospecies biofilms (*S. oralis, A. actinomycetemcomitans*, and *P. gingivalis*) for 3.5 h. On the one hand, neutrophils without PMA (non-activated neutrophils) were used to analyze the induced activation of neutrophils by the respective biofilm. On the other hand, neutrophils were activated with the protein kinase C activator PMA, which is a widely used experimental substance to efficiently stimulate various neutrophil reactions, such as ROS production, NETosis, apoptosis, or degranulation [56–58]. With the activated neutrophils, the capacity of each monospecies biofilm to affect the already activated antimicrobial mechanism of neutrophils was determined. After the incubation, the neutrophil responses were analyzed for NETs release, ROS production, and secretion of MMP-8 and -9.

### NET formation

**Figure 1.**
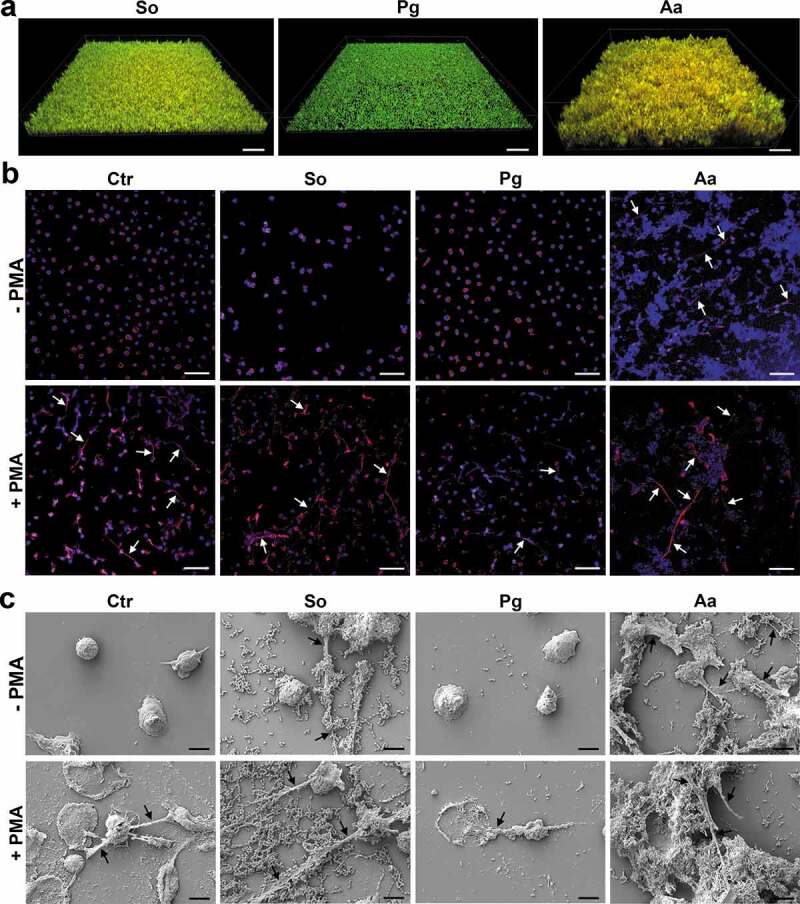
NET formation by neutrophils after challenge with the different biofilms in the presence or absence of PMA. (a) Biofilms before the cultivation with neutrophils. Representative 3D image of different biofilms (*Streptococcus oralis, Porphyromonas gingivalis* and *Aggregatibacter actinomycetemcomitans*) stained by live/dead. Viable bacteria are visualized in green and dead in orange/red. Scale bars: 40 µm. (b) The neutrophils associated with the different biofilms were fixed after incubation for 3.5 h. Immunocytochemistry was performed to visualize cell nuclei (blue; DAPI) and NETs, which were double-stained (red; anti-human MPO antibody and blue; DAPI). No NETs formation, which is illustrated by a clear nuclei-located DAPI-staining and MPO signal closely surrounding the nuclei, was observed in untreated neutrophils. NETs formation indicated by arrows, was observed after cultivation with *A. actinomycetemcomitans* biofilm in absence of PMA as well as in all samples treated with PMA. Representative images are shown. Scale bars: 50 µm. (c) The samples were fixed, dried, and sputtered after incubation. Representative SEM images from four donors show the bacteria, neutrophils and any formed NETs, which are indicated by arrows. Scale bars: 5 µm; Ctr = control without biofilm; So = *S. oralis*; Pg = *P. gingivalis*; Aa = *A. actinomycetemcomitans.*

Neutrophils were analyzed for their NET release using immune fluorescence staining (red; anti-human MPO antibody and blue; DAPI) and SEM (scanning electron microscopy). In the absence of PMA, untreated neutrophils do not form NETs illustrated by a clear nuclei-located DAPI-staining and MPO signal closely surrounding the nuclei (Figure 1(b)) and demonstrated in the SEM picture (Figure 1(c)), too. *A. actinomycetemcomitans* stimulated NET formation, which was visualized by co-staining of wide-spread DNA and the NET marker MPO (Figure 1(b)) and in the SEM picture (Figure 1(c)). Additionally, NET formation was also observed after cultivation with *S. oralis* (Figure 1(b,c)); however, it seemed to be that *A. actinomycetemcomitans* induced a stronger NET formation. In contrast, no NETs were detectable after cultivation with *P. gingivalis* (Figure 1(b,c)).

In the presence of PMA, NETs were detected in the control without biofilm as well as in the *S. oralis* and in the *A. actinomycetemcomitans* samples. Compared to the PMA only-treated cells, the incubation of the neutrophils with *P. gingivalis* appeared to show slightly less NETs (Figure 1(b,c)). Noteworthy, observations from immune fluorescence staining regarding NETs formation could be confirmed by SEM.

### Nuclease activity

**Figure 2.**
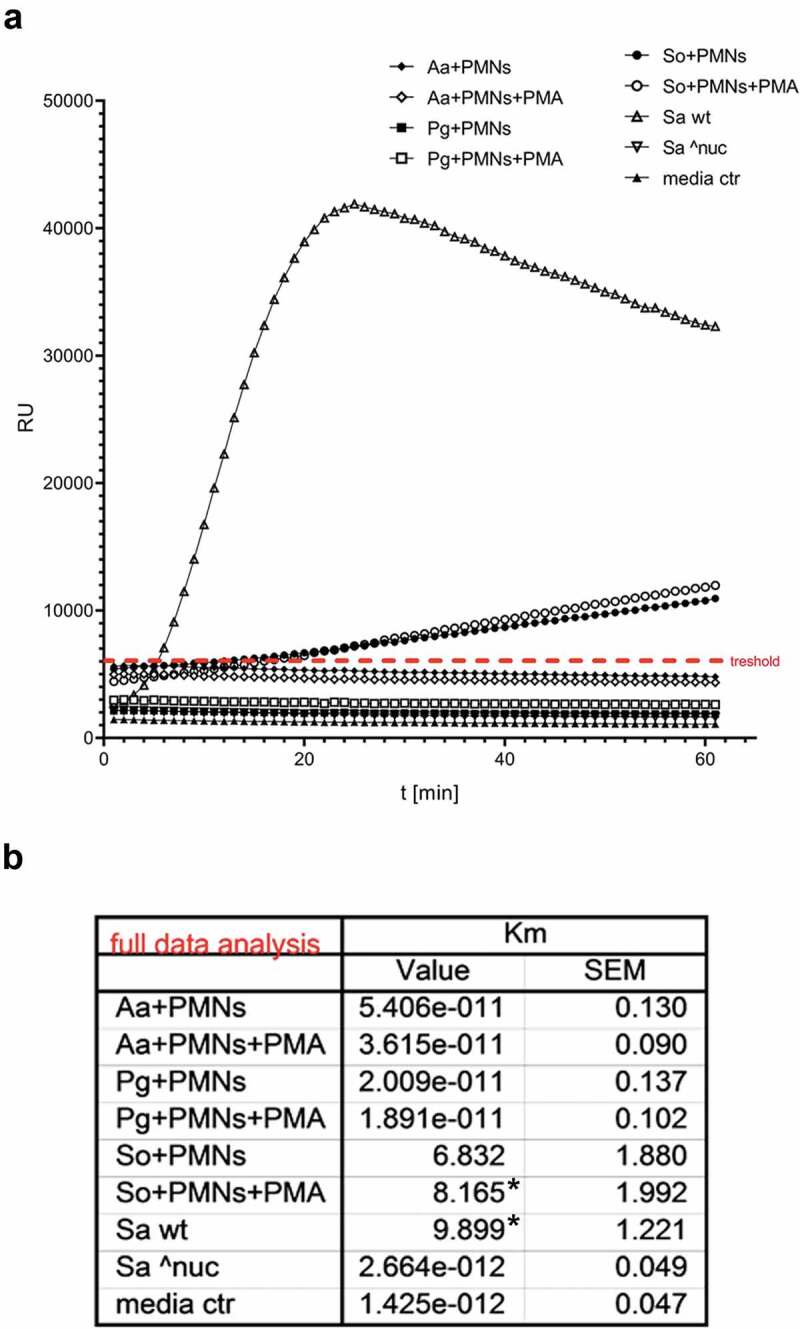
Nuclease activity in the collected supernatants after 3.5 h cultivation of the neutrophils with the different biofilms. (a) The graph shows the typical kinetics of the nuclease assay for 60 min. Km values were determined in relation to the threshold value (red line) and (b) the mean values of four samples are listed in the table. Statistical differences to *S. aureus* nuclease knock-out (Sa^nuc) were analyzed using one-way ANOVA with Bonferroni correction and were considered significant at *p < 0.05. Aa = *A. actinomycetemcomitans*; Pg = *P. gingivalis*; So = *S. oralis*; Sa wt = *S. aureus* wildtype; Sa ^nuc = *S. aureus* nuclease knock-out

As a response to formation of NETs, several bacteria are known to produce nuclease to degrade NETs and avoid entrapment and/or killing by NETs [59,60]. Therefore, the nuclease activity was investigated in the supernatants after co-cultivation for 3.5 h using a DNA molecular beacon and compared to *S. aureus* nuclease knock-out (Sa^nuc) (Figure 2). Groups with *A. actinomycetemcomitans* and *P. gingivalis* showed no nuclease activity, neither with nor without PMA treatment. However, significant nuclease activity was detected in the *S. oralis* group with PMA treatment.

### ROS production

**Figure 3.**
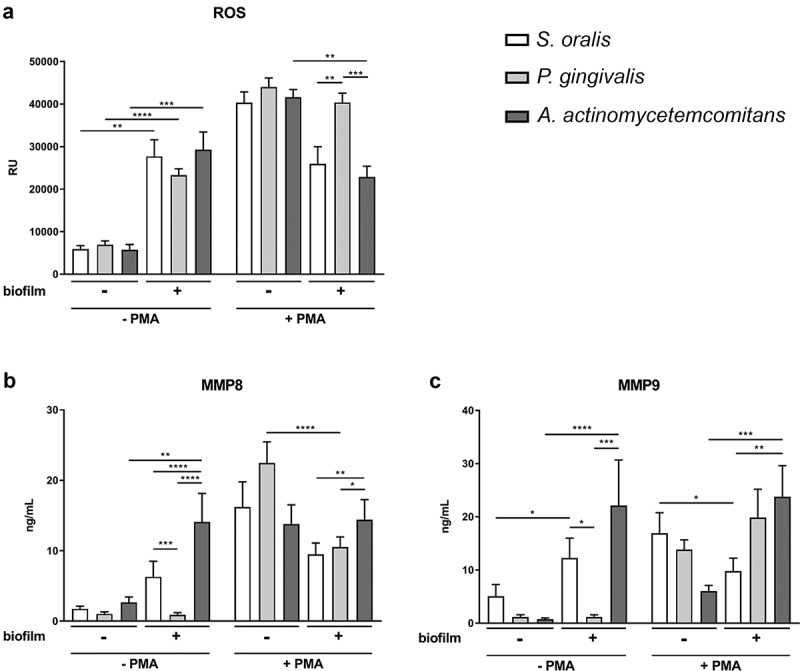
ROS production and MMP secretion of neutrophils in response to the different biofilms in the presence or absence of PMA. (a) ROS was determined for 3 h by DCF-DA and means (± SEM) of the 3 h time point are shown in the bar graph. (b) MMP-8 and (c) MMP-9 were measured by ELISAs in the supernatants collected after biofilm incubation for 3.5 h. The bar graphs represent the means (± SEM). ROS production and MMP secretion were determined from four donors in technical triplicates. Differences between the groups were analyzed using two-way ANOVA with Bonferroni correction. *p < 0.05; **p < 0.01; ***p < 0.001; ****p < 0.0001

ROS levels were determined during the incubation with the different biofilms in the presence or absence of PMA (Figure 3(a) and Figure S1). The ROS kinetics showed an almost constant ROS level during the whole incubation in the respective treatment groups (Figure S1). After 3.5 h co-cultivation (Figure 3(a)), all three biofilms significantly increased ROS production in the absence of PMA. PMA-treatment resulted in ROS production as well [56]; however, *A. actinomycetemcomitans* significantly decreased the ROS level of PMA-stimulated neutrophils compared to the control without biofilm, and the same trend was observed for *S. oralis*.

### MMP secretion

MMP-8 and MMP-9 levels were determined in the supernatant after co-cultivation for 3.5 h using ELISA (Figure 3(b,c)). In the absence of PMA, *A. actinomycetemcomitans* significantly increased the secretion of MMP-8 and -9, and *S. oralis* increased the secretion of MMP-9. The co-incubation with *P. gingivalis* showed a similar level of MMP-8 and MMP-9 compared to the control without biofilm.

After activation with PMA, *P. gingivalis* significantly reduced the MMP-8 levels and *S. oralis* the MMP-9 levels. *A. actinomycetemcomitans* significantly increased the MMP-9 secretion – despite PMA activation. Additionally, the amount of MMP-8 and MMP-9 release after cultivation with *A. actinomycetemcomitans* was similar in the presence or absence of PMA.

## Discussion

Neutrophils are the main innate immune cells in the oral cavity and are important for the host’s response to oral biofilm [22,23]. The balance in function and number of neutrophils is crucial for oral health [21,22]. The imbalance can contribute to tissue destruction which can lead to periodontitis [30]. Neutrophils are able to respond specifically and differently through their antimicrobial mechanisms to various bacterial species [42] and this might play an important role for oral health or diseases development. Therefore, in order to further explore this role of neutrophils, the aim was to investigate the bacterial species-specific effects on the antimicrobial mechanisms of neutrophils by commensal (*S. oralis*) and pathogenic (*P. gingivalis* or *A. actinomycetemcomitans*) monospecies biofilms.

The isolated human neutrophils were incubated with the different biofilms for 3.5 h. On the one hand, the activation of neutrophils by the respective biofilm was investigated using unstimulated neutrophils. On the other hand, modification especially the inhibition of antimicrobial mechanism of neutrophils by the respective biofilm was analyzed using PMA-activated neutrophils. In order to answer the question whether the bacterial biofilms are able to inhibit the antimicrobial defense mechanisms of neutrophils, a high level of stimulation was required. For this reason, PMA as a strong stimulus of the neutrophil response, which is extensively described as such in the literature [52] and worldwide used in laboratories, was used despite the fact that it is a non-physiological stimulus. As selected neutrophil responses, NETs release, ROS production, and secretion of MMP-8 and -9 were analyzed. NET formation was depicted in representative images since the three-dimensional structure, and the considerable background fluorescence from the bacterial DNA, significantly prevents the possibility of NET-quantification, e.g. using ImageJ. NET-quantification in a 3D structure, as in the case of the bacterial biofilms, has not yet been performed successfully [61]. Therefore, NET formation was analyzed by two different imaging methods to get more accurate results. The 3.5 h time point was chosen, since only the highest possible activity of the neutrophils enables to analyze the effect (especially inhibition) on the antimicrobial mechanisms by the bacterial biofilms. Especially, for the analysis of the NET formation, this is the commonly used time point [44,53,58]. The presented data for ROS production supported the results of NETs formation. The results for the individual biofilms showed up at an early stage which was depicted in the ROS-kinetics (Figure S1). Since MMPs concentrated in the test sample due to the continuous secretion, the late 3.5 h time point was also chosen for MMP release analysis. As summarized in Table 1, specific responses of neutrophils to different biofilms were detectable, and this was reflected in all investigated antimicrobial mechanisms: NET formation, ROS production and MMP-8 and -9 releases. Generally, a species-specific response of neutrophils was observed, which is in accordance with previous studies [42,44].

**Table 1.** Evaluation of the antimicrobial response of neutrophils to the different biofilms in the absence or presence of PMA

	Control		*S. oralis*		*P. gingivalis*		*A. actinomycetemcomitans*	
	- PMA	+ PMA	- PMA	+ PMA	- PMA	+ PMA	- PMA	+ PMA
**NETs**	ND	D	D	D	ND	D	D	D
**ROS**	-	+++	++	++	+++	+++	+++	++
**MMP-8**	-	+++	+	+	-	+	+++	+++
**MMP-9**	-	++	+	+	-	++	+++	+++

- no/slight neutrophil response; + moderate neutrophil response; ++ high neutrophil response; +++ excessive neutrophil response; ND not detected; D detected

### S. oralis

The commensal *S. oralis* monospecies biofilm slightly enhanced NET formation in the untreated neutrophils, in accordance with the results of Oveisi et al. [44]. NETs protect the host against bacterial invasion in epithelial cells [23] and are important for the maintenance of periodontal health [28]. Therefore, the NETs enhancement induced by commensals like *S. oralis* might correspond to protective activation of neutrophils as a part of a healthy innate immune response [44]. On the other hand, *S. oralis* slightly suppressed NETs in PMA-activated neutrophils. This may be explained by the nuclease activity of *S. oralis*, which is in accordance with previous studies [59,60]. The degradation of the NET structure by commensals may contribute to removal of excessive NETs. This phenomenon might support oral health, as impaired NET clearance leads to a high concentration of NET-bound proteins and these support tissue destruction [29]. Thus, it seems that *S. oralis* is able to induce both release and degradation of NETs and thereby it might support a balance in the level of NETs, which is necessary for oral health [28,29].

The *S. oralis* biofilm increased ROS production in the absence of PMA and showed a tendency to reduce it after PMA activation. Thereby, after cultivation with *S. oralis*, ROS levels were similar in the PMA-activated as well as in neutrophils without PMA. A previous study showed that *S. oralis* induces activation of a transcription factor for antioxidants (nuclear factor erythroid 2-related factor, Nrf2) in neutrophils in parallel with induced ROS production [44]. The resulting antioxidants from neutrophils neutralize ROS [62] and may reduce the ROS, as we observed in our study. Thus, *S. oralis* induced ROS production but might limit the damage in case of an excess ROS level by generation of antioxidants in the same immune cell [44]. Similar to NETs, this balance of ROS might be important to maintain oral health.

*S. oralis* biofilm increased the secretion of MMP-9 in the absence of PMA, but after PMA activation, the MMP-9 level was reduced to the same level as observed without PMA. The same trend was also observed for MMP-8. Hence, *S. oralis* created a balance of MMP-8 and -9 levels, as we observed also for NETs and ROS. The balance of MMPs is important for maintaining oral health because both absence and enhancement of MMP secretion contribute to the progression of periodontitis [35,36,38]. Taken together, our results demonstrated that the *S. oralis* biofilm modulated neutrophils in a way that the tissue destructive responses of neutrophils could be balanced, which might be important to maintain oral health.

### P. gingivalis

The pathogen *P. gingivalis* monospecies biofilm seemed to reduce NETs after PMA activation and no NETs were detected in the absence of PMA. The reduction in NETs may be mediated by bacterial DNases [42,60]. The used *P. gingivalis* strain was shown to have a slight DNase activity [59]. However, this was not reflected in our results. It is possible that *P. gingivalis* DNase degraded the NETs directly after starting the incubation with the neutrophils, as NET degradation is a rapid process and may occur within 90 min [60]. During the incubation for 3.5 h, DNases were perhaps degraded by gingipains [60] and could therefore not be measured in the nuclease activity assay at the end of the incubation. The expression of bacterial DNase and degradation of NETs might support the bacterial invasion of *P. gingivalis* in the tissue [63]. The evasion of host defenses may lead to increased pathogenicity and might cause periodontitis [28].

*P. gingivalis* biofilm induced ROS production in the absence of PMA and there was no reduction in ROS after PMA activation. These observations correspond to clinical studies in which neutrophils from chronic periodontitis patients (according to the old classification) produced higher levels of ROS compared to healthy patients [64]. Bacteria possess defense mechanisms against oxidative stress. The expression of ruberythrin by *P. gingivalis* protects both *P. gingivalis* and other biofilm organisms from oxidative stress [42,65]. Thus, the elevated release of ROS can cause tissue destruction, whereas the biofilm remains protected. Both together play an important role in the pathogenesis of periodontitis and the associated tissue destruction [66].

After incubation with *P. gingivalis* in absence of PMA, low levels of MMP-8 and -9 were detected which were similar to controls without biofilm. Moreover, MMP-8 was reduced by *P. gingivalis* after PMA stimulation. These low levels of MMPs (with and without PMA) may have been caused by a suppression of the MMP release or by gingipains during the incubation of 3.5 h. These proteases are one of the main virulence factors of *P. gingivalis* and can cleave many host components, such as cytokines, immunoglobulins, complement factors, MMPs, or DNases, as discussed above, to overcome the host defense mechanism [40,67]. Taken together, NET formation and MMP-8 and -9 secretions were diminished by *P. gingivalis*, probably to evade antimicrobial mechanisms of neutrophils. Nevertheless, ROS levels were high for which *P. gingivalis* has a protection system. Additionally, *P. gingivalis* was shown to exhibit various mechanisms in order to evade neutrophil-mediated killing, which may contribute to its colonization and invasion in the tissue [40,63]. Consequently, neutrophils may not be able to clear this pathogen. The continuous presence of the pathogen may lead to persistent neutrophil recruitment and activation in a later stage of infection, which subsequently may cause hyper-activation of neutrophils leading to inflammation with tissue destruction providing nutrients for *P. gingivalis* [40,68]. Therefore, periodontitis might be actively supported by continuing neutrophil reaction to *P. gingivalis*, which causes parallel damage to host tissue. This type of neutrophil activation might be accompanied by a slow progression of periodontitis since only some tissue-destructive antimicrobial mechanisms were induced.

### A. actinomycetemcomitans

The pathogen *A. actinomycetemcomitans* monospecies biofilm induced strong NETs formation, in accordance with previous studies [29,30,69]. This may be caused by the virulence factor leukotoxin (Ltx) [30]. The *A. actinomycetemcomitans* JP2 strain, which was used in our study, expresses constant high levels of Ltx [70]. Ltx lyses leukocytes – but also activates neutrophils by induction of migration, degranulation, and NETosis, which is independent of and appears before Ltx-mediated lysis [30,71,72]. The NETs were not degraded, which could be explained by the missing nuclease activity of *A. actinomycetemcomitans*, which is in line with findings of Doke et al. [60]. The exaggerated NETs formation, which is often found in an aggressive form of periodontitis, can cause tissue destruction by NET-bound proteins and enzymes, such as neutrophil elastase or MPO and concomitant release of cytotoxic molecules and has a crucial role in the pathogenesis of periodontitis [28–30].

ROS production was increased by *A. actinomycetemcomitans* in the absence of PMA, which may be caused by Ltx binding on neutrophils [30]. However, ROS level of PMA-activated neutrophils was reduced by *A. actinomycetemcomitans*. This bacterium has two mechanisms to avoid the toxic effect of ROS. In response to oxidative stress, specific genes in *A. actinomycetemcomitans* are upregulated to neutralize ROS (*katA*) or to escape from the toxic environment (*dspB*) [18]. This may explain the decreased ROS level after PMA activation. In contrast to *S. oralis, A. actinomycetemcomitans* has not the ability to induce the transcription factor Nrf2 [44] and thereby the production of antioxidants in neutrophils. Consequently, the important balance between ROS and antioxidants of neutrophils, which protect the tissue, may be disrupted [32]. This imbalance and the excessive level of ROS are closely associated with aggressive periodontitis [73].

MMP-8 and -9 secretions were markedly increased by the *A. actinomycetemcomitans* biofilm. The excessive secretion of MMP-8 and -9 leads to the degradation of collagen in the soft tissue and the alveolar bone and are associated with tissue destruction during periodontitis [74]. The high concentration of MMP-8 has been linked to the severity of inflammation in clinical studies and was associated with an aggressive form of periodontitis [37,75]. Taken together, the *A. actinomycetemcomitans* biofilm induced a severe neutrophil response by the release of NETs, ROS, and MMPs, which may lead to tissue destruction and contribute to the pathogenesis of periodontitis. Thus, this immediate hyperactive reaction of the neutrophils might contribute to a rapid progression of periodontitis, since different tissue-destructive antimicrobial mechanisms were induced.

Our results demonstrated that the neutrophil response was different to each species and the type of response could be associated with possible contribution to oral health or periodontitis. It should be considered that the use of monospecies biofilms does not optimally mimic the clinical situation, where a multispecies biofilm always exists [5,6]. However, in order to clarify the role of different bacterial species in the native multispecies biofilm and the specific neutrophil response to these species, it is necessary to investigate the interaction with one bacterial species alone. Compared to planktonic bacteria, the use of monospecies biofilms is one step closer to the clinical situation, as biofilm is the common way of life in the oral cavity and bacteria within the biofilm are more resistant to the host’s immune response [5,8,9,45].

## Conclusion

In conclusion, the neutrophil responses, NET formation, ROS production and MMPs secretion were different to the various bacterial species grown as monospecies biofilm. The commensal *S. oralis* induced a balance-like neutrophil response – which is consistent with the concept that commensals protect the host against the overgrowth of pathogenic bacteria and are required to maintain oral health [23,76]. The pathogenic biofilms induced different neutrophil responses. *P. gingivalis* partially diminished the neutrophil response, which might support its colonization and invasion of the tissue leading to inflammation. These might contribute to the pathogenesis of periodontitis and might be accompanied with a slow progression. In contrast, *A. actinomycetemcomitans* induced a strong neutrophil reaction, which might cause tissue destruction and perhaps contributes to a more rapid progression of periodontitis. Thus, the different neutrophil responses and the occurrence of these bacterial species in the native multispecies biofilm might contribute to either the maintenance of oral health or the pathogenesis of periodontal disease. These findings and future investigations may provide new opportunities for future strategies for disease treatment, where immunomodulatory drugs could be one option.

## Supplementary Material

Supplemental Material

## References

[1] Krishnan K, Chen T, Paster BJ. A practical guide to the oral microbiome and its relation to health and disease. Oral Dis. 2017 Apr;23(3):276–12. 27219464 10.1111/odi.12509 PMC5122475

[2] Mosaddad SA, Tahmasebi E, Yazdanian A, et al. Oral microbial biofilms: an update. Eur J Clin Microbiol Infect Dis. 2019 Nov;38(11):2005-2019. 10.1007/s10096-019-03641-9 31372904

[3] Zaura E, Keijser BJ, Huse SM, et al. Defining the healthy “core microbiome” of oral microbial communities. BMC Microbiol. 2009 Dec 15;9:259–2180-9-259. 10.1186/1471-2180-9-259 PMC2805672 20003481

[4] Paster BJ, Olsen I, Aas JA, et al. The breadth of bacterial diversity in the human periodontal pocket and other oral sites. Periodontol. 2000;42:80–87. 10.1111/j.1600-0757.2006.00174.x 16930307

[5] Kolenbrander P, Palmer R, Periasamy S, et al. Oral multispecies biofilm development and the key role of cell-cell distance. Nat Rev Microbiol. 2010;8(7):471–480. 20514044 10.1038/nrmicro2381

[6] Expanded Human Oral Microbiome Database. 2019. [cited 2019 Oct 15]. Available from: http://www.homd.org

[7] Gupta P, Sarkar S, Das B, et al. Biofilm, pathogenesis and prevention–a journey to break the wall: a review. Arch Microbiol. 2016 Jan;198(1):1–15. 26377585 10.1007/s00203-015-1148-6

[8] Hojo K, Nagaoka S, Ohshima T, et al. Bacterial interactions in dental biofilm development. J Dent Res. 2009 Nov;88(11):982–990. 19828884 10.1177/0022034509346811

[9] Flemming H, Wingender J. The biofilm matrix. Nat Rev Microbiol. 2010;8(9):623–633. 20676145 10.1038/nrmicro2415

[10] Hajishengallis G. Periodontitis: from microbial immune subversion to systemic inflammation. Nat Rev Immunol. 2015 Jan;15(1):30–44. 25534621 10.1038/nri3785 PMC4276050

[11] Jenkinson HF, Lamont RJ. Oral microbial communities in sickness and in health. Trends Microbiol. 2005 Dec;13(12):589–595. 16214341 10.1016/j.tim.2005.09.006

[12] Diaz PI, Chalmers NI, Rickard AH, et al. Molecular characterization of subject-specific oral microflora during initial colonization of enamel. Appl Environ Microbiol. 2006 Apr;72(4):2837–2848. 16597990 10.1128/AEM.72.4.2837-2848.2006 PMC1449052

[13] Tonetti MS, Jepsen S, Jin L, et al. Impact of the global burden of periodontal diseases on health, nutrition and wellbeing of mankind: A call for global action. J Clin Periodontol. 2017 May;44(5):456–462. 28419559 10.1111/jcpe.12732

[14] Papapanou PN, Sanz M, Buduneli N, et al. Periodontitis: consensus report of workgroup 2 of the 2017 world workshop on the classification of periodontal and peri-implant diseases and conditions. J Periodontol. 2018 Jun;89(Suppl 1):S173–S182. 29926951 10.1002/JPER.17-0721

[15] Tonetti MS, Greenwell H, Kornman KS. Staging and grading of periodontitis: framework and proposal of a new classification and case definition. J Periodontol. 2018 Jun;89(Suppl 1):S159–S172. 29926952 10.1002/JPER.18-0006

[16] Slots J. Periodontology: past, present, perspectives.Periodontol. 2000 [2013 Jun];62(1):7–19. 10.1111/prd.12011 23574461

[17] Van der Velden U. What exactly distinguishes aggressive from chronic periodontitis: is it mainly a difference in the degree of bacterial invasiveness? Periodontol. 2000 [2017 Oct];75(1):24–44. 10.1111/prd.12202 28758297

[18] Fine DH, Patil AG, Velusamy SK. Aggregatibacter actinomycetemcomitans (Aa) under the radar: myths and Misunderstandings of Aa and its role in aggressive periodontitis. Front Immunol. 2019 Apr;16(10):728. 10.3389/fimmu.2019.00728 PMC6476972 31040843

[19] Ryder MI. Comparison of neutrophil functions in aggressive and chronic periodontitis.Periodontol. 2000 [2000 Jun];53(1):124–137. 10.1111/j.1600-0757.2009.00327.x 20403109

[20] Kantarci A, Oyaizu K, Van Dyke TE. Neutrophil-mediated tissue injury in periodontal disease pathogenesis: findings from localized aggressive periodontitis. J Periodontol. 2003 Jan;74(1):66–75. 12593599 10.1902/jop.2003.74.1.66

[21] Cortes-Vieyra R, Rosales C, Uribe-Querol E. Neutrophil functions in periodontal homeostasis. J Immunol Res. 2016;2016:1396106. 27019855 10.1155/2016/1396106 PMC4785262

[22] Uriarte SM, Edmisson JS, Jimenez-Flores E. Human neutrophils and oral microbiota: a constant tug-of-war between a harmonious and a discordant coexistence. Immunol Rev. 2016 Sep;273(1):282–298. 27558341 10.1111/imr.12451 PMC5353849

[23] Hirschfeld J. Dynamic interactions of neutrophils and biofilms. J Oral Microbiol. 2014 Dec;17(6):26102. 10.3402/jom.v6.26102 PMC4270880 25523872

[24] Kolaczkowska E, Kubes P. Neutrophil recruitment and function in health and inflammation. Nat Rev Immunol. 2013 Mar;13(3):159–175. 23435331 10.1038/nri3399

[25] Rijkschroeff P, Loos BG, Nicu EA. Oral polymorphonuclear neutrophil contributes to oral health. Curr Oral Health Rep. 2018;5(4):211–220. 30524928 10.1007/s40496-018-0199-6 PMC6244624

[26] Krautgartner WD, Vitkov L. Visualization of neutrophil extracellular traps in TEM. Micron. 2008 Jun;39(4):367–372. 17498964 10.1016/j.micron.2007.03.007

[27] Vitkov L, Klappacher M, Hannig M, et al. Extracellular neutrophil traps in periodontitis. J Periodontal Res. 2009 Oct;44(5):664–672. 19453857 10.1111/j.1600-0765.2008.01175.x

[28] Vitkov L, Hartl D, Minnich B, et al. Janus-faced neutrophil extracellular traps in periodontitis. Front Immunol. 2017 Oct;26(8):1404. 10.3389/fimmu.2017.01404 PMC5662558 29123528

[29] White PC, Chicca IJ, Cooper PR, et al. Neutrophil extracellular traps in periodontitis: a web of intrigue. J Dent Res. 2016 Jan;95(1):26–34. 26442948 10.1177/0022034515609097

[30] Hirschfeld J, Roberts HM, Chapple IL, et al. Effects of Aggregatibacter actinomycetemcomitans leukotoxin on neutrophil migration and extracellular trap formation. J Oral Microbiol. 2016 Nov;8(8):33070. 27834173 10.3402/jom.v8.33070 PMC5103672

[31] Magan-Fernandez A, O’Valle F, Abadia-Molina F, et al. Characterization and comparison of neutrophil extracellular traps in gingival samples of periodontitis and gingivitis: A pilot study. J Periodontal Res. 2019 Jun;54(3):218–224. 30298590 10.1111/jre.12621

[32] Wang Y, Andrukhov O, Rausch-Fan X. Oxidative stress and antioxidant system in periodontitis. Front Physiol. 2017 Nov;13(8):910. 10.3389/fphys.2017.00910 PMC5693842 29180965

[33] Pham CT. Neutrophil serine proteases: specific regulators of inflammation. Nat Rev Immunol. 2006 Jul;6(7):541–550. 16799473 10.1038/nri1841

[34] Lin M, Jackson P, Tester AM, et al. Matrix metalloproteinase-8 facilitates neutrophil migration through the corneal stromal matrix by collagen degradation and production of the chemotactic peptide Pro-Gly-Pro. Am J Pathol. 2008 Jul;173(1):144–153. 18556780 10.2353/ajpath.2008.080081 PMC2438292

[35] Dejonckheere E, Vandenbroucke RE, Libert C. Matrix metalloproteinase8 has a central role in inflammatory disorders and cancer progression. Cytokine Growth Factor Rev. 2011 Apr;22(2):73–81. 21388856 10.1016/j.cytogfr.2011.02.002

[36] Gursoy UK, Kononen E, Tervahartiala T, et al. Molecular forms and fragments of salivary MMP-8 in relation to periodontitis. J Clin Periodontol. 2018 Dec;45(12):1421–1428. 30341955 10.1111/jcpe.13024

[37] Boelen GJ, Boute L, d’Hoop J, et al. Matrix metalloproteinases and inhibitors in dentistry. Clin Oral Investig. 2019 Jul;23(7):2823–2835. 10.1007/s00784-019-02915-y 31093743

[38] Kuula H, Salo T, Pirila E, et al. Local and systemic responses in matrix metalloproteinase 8-deficient mice during Porphyromonas gingivalis-induced periodontitis. Infect Immun. 2009 Feb;77(2):850–859. 19029300 10.1128/IAI.00873-08 PMC2632031

[39] Kobayashi SD, Malachowa N, DeLeo FR. Influence of microbes on neutrophil life and death. Front Cell Infect Microbiol. 2017 May;1(7):159. 10.3389/fcimb.2017.00159 PMC5410578 28507953

[40] Sochalska M, Potempa J. Manipulation of neutrophils by porphyromonas gingivalis in the development of periodontitis. Front Cell Infect Microbiol. 2017 May;23(7):197. 10.3389/fcimb.2017.00197 PMC5440471 28589098

[41] Silva LM, Brenchley L, Moutsopoulos NM. Primary immunodeficiencies reveal the essential role of tissue neutrophils in periodontitis. Immunol Rev. 2019 Jan;287(1):226–235. 30565245 10.1111/imr.12724 PMC7015146

[42] Hirschfeld J, White PC, Milward MR, et al. Modulation of neutrophil extracellular trap (NET) and reactive oxygen species (ROS) release by periodontal bacteria. Infect Immun. 2017 Nov 17;85(12). DOI:10.1128/IAI.00297-17. PMC5695129 28947649

[43] van Winkelhoff AJ, Loos BG, van der Reijden WA. van der Velden U. Porphyromonas gingivalis, Bacteroides forsythus and other putative periodontal pathogens in subjects with and without periodontal destruction. J Clin Periodontol. 2002 Nov;29(11):1023–1028. 12472995 10.1034/j.1600-051x.2002.291107.x

[44] Oveisi M, Shifman H, Fine N, et al. Novel assay to characterize neutrophil responses to oral biofilms. Infect Immun. 2019 Jan 24;87(2). Print 2019 Feb. DOI:10.1128/IAI.00790-18. PMC6346121 30455195

[45] Huang R, Li M, Gregory R. Bacterial interactions in dental biofilm. Virulence. 2011;2(5):435–444. 21778817 10.4161/viru.2.5.16140 PMC3322631

[46] Furugen R, Hayashida H, Saito T. Porphyromonas gingivalis and Escherichia coli lipopolysaccharide causes resistin release from neutrophils. Oral Dis. 2013 Jul;19(5):479–483. 23083402 10.1111/odi.12027

[47] Kido J, Kido R, Suryono KM, et al. Induction of calprotectin release by Porphyromonas gingivalis lipopolysaccharide in human neutrophils. Oral Microbiol Immunol. 2004 Jun;19(3):182–187. 15107070 10.1111/j.0902-0055.2004.00139.x

[48] Hiyoshi T, Domon H, Maekawa T, et al. Aggregatibacter actinomycetemcomitans induces detachment and death of human gingival epithelial cells and fibroblasts via elastase release following leukotoxin-dependent neutrophil lysis. Microbiol Immunol. 2019 Mar;63(3–4):100–110. 30817027 10.1111/1348-0421.12672

[49] Kommerein N, Doll K, Stumpp NS, et al. Development and characterization of an oral multispecies biofilm implant flow chamber model. PLoS One. 2018 May 17;13(5):e0196967. 29771975 10.1371/journal.pone.0196967 PMC5957423

[50] Thomas HB, Moots RJ, Edwards SW, et al. Whose gene is it anyway? The effect of preparation purity on neutrophil transcriptome studies. PLoS One. 2015 Sep 24;10(9):e0138982. 26401909 10.1371/journal.pone.0138982 PMC4581699

[51] Köckritz-Blickwede M, Chow O, Ghochani M, et al. Visualization and functional evaluation of phagocyte extracellular traps. Methods Microbiol. 2010;37:139–160.

[52] Brinkmann V, Reichard U, Goosmann C, et al. Neutrophil extracellular traps kill bacteria. Science. 2004 Mar 5;303(5663):1532–1535. 15001782 10.1126/science.1092385

[53] Branitzki-Heinemann K, Mollerherm H, Vollger L, et al. Formation of neutrophil extracellular traps under low oxygen level. Front Immunol. 2016 Nov;25(7):518. 10.3389/fimmu.2016.00518 PMC5122589 27933059

[54] Tolle C, Riedel J, Mikolai C, et al. Biocompatible coatings from smart biopolymer nanoparticles for enzymatically induced drug release. Biomolecules. 2018 Sep 28;8(4):103. 10.3390/biom8040103 PMC6315368 30274232

[55] Schilcher K, Andreoni F, Uchiyama S, et al. Increased neutrophil extracellular trap-mediated Staphylococcus aureus clearance through inhibition of nuclease activity by clindamycin and immunoglobulin. J Infect Dis. 2014 August 1;210(3):473–482. 24526740 10.1093/infdis/jiu091 PMC4091580

[56] Parker H, Albrett AM, Kettle AJ, et al. Myeloperoxidase associated with neutrophil extracellular traps is active and mediates bacterial killing in the presence of hydrogen peroxide. J Leukoc Biol. 2012 Mar;91(3):369–376. 22131345 10.1189/jlb.0711387

[57] Saito T, Takahashi H, Doken H, et al. Phorbol myristate acetate induces neutrophil death through activation of p38 mitogen-activated protein kinase that requires endogenous reactive oxygen species other than HOCl. Biosci Biotechnol Biochem. 2005 Nov;69(11):2207–2212. 16306704 10.1271/bbb.69.2207

[58] Petretto A, Bruschi M, Pratesi F, et al. Neutrophil extracellular traps (NET) induced by different stimuli: A comparative proteomic analysis. PLoS One. 2019 Jul 8;14(7):e0218946. 31283757 10.1371/journal.pone.0218946 PMC6613696

[59] Palmer LJ, Chapple IL, Wright HJ, et al. Extracellular deoxyribonuclease production by periodontal bacteria. J Periodontal Res. 2012 Aug;47(4):439–445. 22150619 10.1111/j.1600-0765.2011.01451.x

[60] Doke M, Fukamachi H, Morisaki H, et al. Nucleases from Prevotella intermedia can degrade neutrophil extracellular traps. Mol Oral Microbiol. 2017 Aug;32(4):288–300. 27476978 10.1111/omi.12171 PMC5516193

[61] van Breda SV, Vokalova L, Neugebauer C, et al. Computational methodologies for the in vitro and in situ quantification of neutrophil extracellular traps. Front Immunol. 2019 Jul;10(10):1562. 31354718 10.3389/fimmu.2019.01562 PMC6635468

[62] Chiu AV, Saigh MA, McCulloch CA, et al. The role of nrf2 in the regulation of periodontal health and disease. J Dent Res. 2017 Aug;96(9):975–983. 28617616 10.1177/0022034517715007

[63] Pinnock A, Murdoch C, Moharamzadeh K, et al. Characterisation and optimisation of organotypic oral mucosal models to study Porphyromonas gingivalis invasion. Microbes Infect. 2014 Apr;16(4):310–319. 24491281 10.1016/j.micinf.2014.01.004

[64] Chapple IL, Matthews JB. The role of reactive oxygen and antioxidant species in periodontal tissue destruction. Periodontol. 2000;43(1):160–232. 10.1111/j.1600-0757.2006.00178.x 17214840

[65] Mydel P, Takahashi Y, Yumoto H, et al. Roles of the host oxidative immune response and bacterial antioxidant rubrerythrin during Porphyromonas gingivalis infection. PLoS Pathog. 2006 Jul;2(7):e76. 16895445 10.1371/journal.ppat.0020076 PMC1522038

[66] Dahiya P, Kamal R, Gupta R, et al. Reactive oxygen species in periodontitis. J Indian Soc Periodontol. 2013 Jul;17(4):411–416. 24174716 10.4103/0972-124X.118306 PMC3800399

[67] Bondy-Carey JL, Galicia J, Bagaitkar J, et al. Neutrophils alter epithelial response to Porphyromonas gingivalis in a gingival crevice model. Mol Oral Microbiol. 2013 Apr;28(2):102–113. 23193955 10.1111/omi.12008 PMC3594541

[68] de Buhr N, Bonilla MC, Pfeiffer J, et al. Degraded neutrophil extracellular traps promote the growth of Actinobacillus pleuropneumoniae. Cell Death Dis. 2019 Sep 10;10(9):657-019-1895-4. 10.1038/s41419-019-1895-4 PMC6736959 31506432

[69] Palmer LJ, Damgaard C, Holmstrup P, et al. Influence of complement on neutrophil extracellular trap release induced by bacteria. J Periodontal Res. 2016 Feb;51(1):70–76. 25900429 10.1111/jre.12284

[70] Szafranski SP, Deng ZL, Tomasch J, et al. Quorum sensing of Streptococcus mutans is activated by Aggregatibacter actinomycetemcomitans and by the periodontal microbiome. BMC Genomics. 2017 Mar 20;18(1):238-017-3618-5. 10.1186/s12864-017-3618-5 PMC5359896 28320314

[71] Johansson A, Claesson R, Hanstrom L, et al. Polymorphonuclear leukocyte degranulation induced by leukotoxin from Actinobacillus actinomycetemcomitans. J Periodontal Res. 2000 Apr;35(2):85–92. 10863962 10.1034/j.1600-0765.2000.035002085.x

[72] Spitznagel J Jr, Kraig E, Kolodrubetz D. Regulation of leukotoxin in leukotoxic and nonleukotoxic strains of Actinobacillus actinomycetemcomitans. Infect Immun. 1991 Apr;59(4):1394–1401. 2004819 10.1128/iai.59.4.1394-1401.1991 PMC257855

[73] Baltacioglu E, Yuva P, Aydin G, et al. Lipid peroxidation levels and total oxidant/antioxidant status in serum and saliva from patients with chronic and aggressive periodontitis. Oxidative stress index: a new biomarker for periodontal disease? J Periodontol. 2014 Oct;85(10):1432–1441. 24635543 10.1902/jop.2014.130654

[74] Romero-Castro NS, Vazquez-Villamar M, Munoz-Valle JF, et al. Relationship between TNF-alpha, MMP-8, and MMP-9 levels in gingival crevicular fluid and the subgingival microbiota in periodontal disease. Odontology. 2020 Jan;108(1):25-33. doi: 10.1007/s10266-019-00435-5. Epub 2019 Jun 18. 31214897

[75] Lira-Junior R, Ozturk VO, Emingil G, et al. Salivary and serum markers related to innate immunity in generalized aggressive periodontitis. J Periodontol. 2017 Dec;88(12):1339–1347. 28753101 10.1902/jop.2017.170287

[76] Ingendoh-Tsakmakidis A, Mikolai C, Winkel A, et al. Commensal and pathogenic biofilms differently modulate peri-implant oral mucosa in an organotypic model. Cell Microbiol. 2019 July 3 [2019 7];21(10):e13078. 31270923 10.1111/cmi.13078 PMC6771885

