# Supplementary material for: Neutrophils exhibit an individual response to different oral bacterial biofilms
Source: J Oral Microbiol. 2020 Dec 9;13(1):1856565. doi: 10.1080/20002297.2020.1856565 (PMC7733916; doi:10.1080/20002297.2020.1856565)
Supplement: Supplemental Material [file ZJOM_A_1856565_SM8724.docx]

*Neutrophils exhibit an individual response to different oral bacterial biofilms*

Carina Mikolai^1#*^, Katja Branitzki-Heinemann^2#*^, Alexandra Ingendoh-Tsakmakidis^1^, Meike Stiesch^1^, Maren von Köckritz-Blickwede^2^, Andreas Winkel^1^

^1^Department of Prosthetic Dentistry and Biomedical Materials Science, Hannover Medical School, Hanover, Germany

^2^Department of Physiological Chemistry, and Research Center for Emerging Infections and Zoonoses (RIZ), University of Veterinary Medicine Hannover, Germany.

**Supplementary Material**


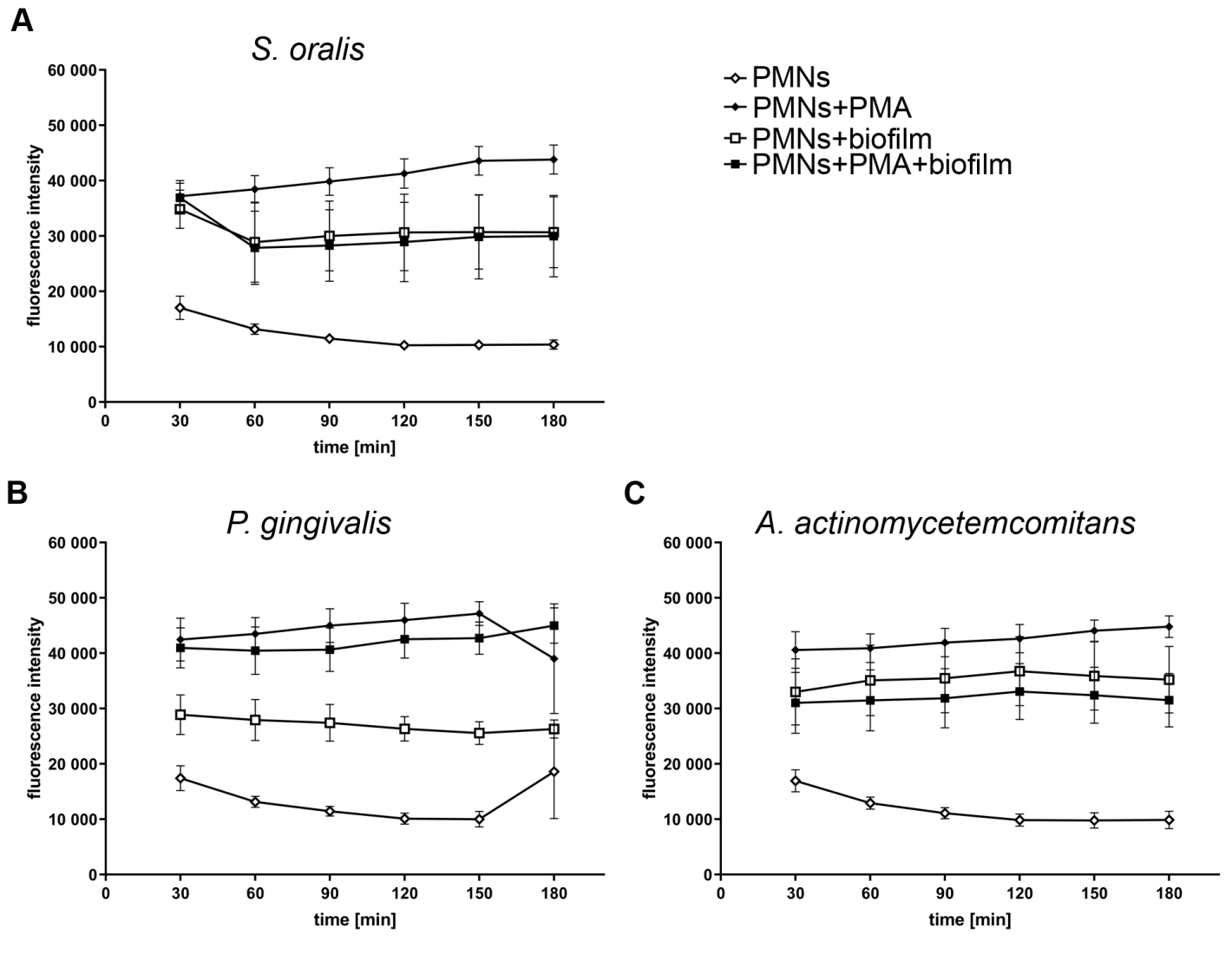


**Figure S1.** ROS production of neutrophils in response to the different biofilms in the presence or absence of PMA. ROS was determined every 30 min over a time period of 3 h by adding DCF-DA. Presented are means ± SEM. (A) *Streptococcus oralis*; (B) *Porphyromonas gingivalis*; (C) *Aggregatibacter actinomycetemcomitans*.
